# “Acute kidney dysfunction with no rejection” is associated with poor renal outcomes at 2 years *post* kidney transplantation

**DOI:** 10.1186/s12882-019-1444-5

**Published:** 2019-07-09

**Authors:** François Paquot, Laurent Weekers, Catherine Bonvoisin, Hans Pottel, François Jouret

**Affiliations:** 1 Division of Nephrology, Department of Internal Medicine, University of Liège Hospital, Avenue Hippocrate, 13 – B4000, Liège, Belgium; 2 KU Leuven Kulak, Department of Public Health and Primary Care, University of Leuven, Kortrijk, Belgium; 3 Groupe Interdisciplinaire de Génoprotéomique Appliquée, Cardiovascular Sciences, University of Liège, Liège, Belgium

**Keywords:** Acute kidney dysfunction with no rejection (ADNR), Kidney transplant recipients (KTR), Estimated glomerular filtration rate (eGFR), Acute rejection (AR), Outcomes

## Abstract

**Background:**

“Acute kidney dysfunction with no rejection” (ADNR) corresponds to acute kidney injury without histological evidence of acute rejection (AR) in kidney transplant recipients (KTR). The prognosis of ADNR is unknown.

**Methods:**

From 2007 to 2015, we categorized KTR with for-cause kidney biopsy within the first 12 months *post* kidney transplantation (KTx) into ADNR (*n* = 93) and biopsy-proven AR (*n* = 22). Controls (C, *n* = 135) included KTR with no ADNR or AR within the first 24 months post-KTx. A piecewise linear regression with a single fixed-knot at 12 months served to establish intercepts and slopes of MDRD-eGFR variations from 12 to 24 months. The percentage of KTR with ≥30% reduction of eGFR from 12 to 24 months was calculated as a surrogate marker of future graft loss.

**Results:**

The median time for for-cause biopsy was 22 [10–70] and 13 [7–43] days for ADNR and AR, respectively. At 12 months, eGFR was significantly higher in C (57.6 ± 14.9 mL/min/1.73m^2^) vs. ADNR (43.5 ± 15.4 mL/min/1.73m^2^, *p* < 0.0001) and vs. AR (46.5 ± 15.2 mL/min/1.73m^2^, *p* < 0.0065). The proportion of KTR with ≥30% reduction in eGFR from 12 to 24 months reached 16.3% in C vs. 29.9% in ADNR (*p* = 0.02) and vs. 15% in AR (not significant).

**Conclusions:**

ADNR is associated with poor outcomes within 2 years post-KTx.

## Background

Kidney transplantation (KTx) is currently the best therapeutic option for patients suffering from end-stage renal disease, with reduced all-cause and cardiovascular mortality and improved quality of life as compared to chronic dialysis [1]. Still, acute rejection (AR) undermines the full benefits of KTx [2, 3]. Typically, AR is suspected when serum creatinine (SCr) rises from individual baseline value [4]. Although various non-invasive approaches are currently under development [2, 5–10], the gold standard for AR diagnosis still relies on kidney graft biopsy and Banff classification [11–13]. However, a significant number of KTR presenting with acute elevation of SCr show a normal renal histology, with no biopsy-proven AR. Kurian S.M. and colleagues introduced the concept of “acute dysfunction with no rejection” (ADNR) in 2014 [10]. To the best of our knowledge, there is no data concerning the long-term outcomes of KTR with ADNR, in comparison to (i) KTR with biopsy-proven AR or (ii) KTR with no acute kidney injury (AKI) during the follow-up.

In 2016, Clayton P.A. et al. [14] demonstrated that ≥30% decline in estimated glomerular filtration rate (eGFR) between 12 and 24 months post-KTx is strongly associated with risks of subsequent death and death-censored graft failure among KTR, which advocates for the use of percentage decline in eGFR as a surrogate outcome in KTx studies.

Here, we calculated the eGFR at 12 months post-KTx and the eGFR ≥30% decline between 12 and 24 months post-KTx, in KTR with ADNR versus biopsy-proven AR and controls (C).

## Methods

### Patients

All KTx performed from January 2007 to June 2015 were retrieved from our prospective database at the University of Liège Hospital (ULiège CHU) in Liège, Belgium. KTx with primary non-function or BK virus nephropathy were excluded. Kidney graft biopsies were graded according to the Banff 2013 classification [13]. Histological lesions were scored as discrete variables (from 0 to 3) on the basis of leukocyte infiltration severity in each component: glomeruli (g), peritubular capillaries (ptc), arteries (v), tubules (t), and interstitium (i). Biopsies were considered normal when there were no tubulitis (t = 0), no vasculitis (v = 0), no or mild microvascular inflammation (g + ptc < 2) without circulating donor specific antibody (DSA) and no features of a disease process. Borderline changes were defined as mild to severe tubulitis (t > 0) with no to severe interstitial inflammation (i0–3) not reaching the threshold for acute cellular rejection definition, and no feature of a specific disease process. Biopsies diagnosed as cellular AR were defined as having a Banff score ≥ i2 and ≥ t2 and/or v > 0. Antibody mediated AR definition required three criteria: circulating DSA, micro- (g and/or ptc > 0) or macro- vascular inflammation (v > 0) and evidence of antibody interaction with the endothelium either as c4d deposition or as at least moderate microvascular inflammation (g + ptc > =2). All the included biopsies met adequacy criteria (at least 7 glomeruli and 1 artery) [12]. All the biopsies performed during the first 12 months post-KTx were extracted from the database, as well as all individual creatinine values, and these results were used to categorize the patients in 2 groups, i.e. AR and ADNR. All the for-cause biopsies were clinically indicated. When a KTR received several biopsies in that time frame, the first adequate graft biopsy within the first 12 months post-KTx was kept for analysis. The ADNR group included patients with no histological evidence of AR. The AR group included KTR with biopsy-proven AR. The control (C) group included KTR with no AKI over a 24-month follow-up post-KTx and normal renal histology at 3 months post-KTx (protocol biopsy).

### Statistics

The eGFR was determined using the Modification of Diet in Renal Disease (MDRD) Study equation [15]. MDRD is based on SCr upon the following formula: 175 x SCr (mg/dl)^-1.154^ x age^-0.203^ × 0.742 (if woman). C*ontinuous piecewise linear regression* with a fixed *knot* at 12 months for eGFR versus time were fitted to generate two regression lines, i.e. one corresponding to the first 12 months post-KTx and one corresponding to the follow-up between 12 and 24 months post-KTx. The linear regressions were calculated individually for each patient and the slopes of the second part of the regression were used to describe the evolution of eGFR over that time-frame. The baseline eGFR value (eGFR1Y) was taken as the predicted value at the knot (12 months). Likewise, the final eGFR value (eGFR2Y) was calculated as the value predicted by the second linear regression at 24 months exactly. Percentage decline of eGFR between 12 and 24 months post-KTx was calculated as (eGFR2Y – eGFR1Y)/eGFR1Y * 100. Finally, the proportion of KTR showing a ≥ 30% reduction of eGFR between 12 and 24 months post-KTx was determined in each group [14, 16]. Evidence that regressions differed among groups was obtained by comparing eGFR intercepts and percentage decline. Analysis of variance (ANOVA) or χ^2^ test (Fisher’s Exact test for 2 × 2 tables) were used, as appropriate, to compare the clinical characteristics of patients belonging to the 3 groups. ANOVA was followed by a post hoc Tukey’s Studentized Range test correcting for multiple comparisons. All analyses were done with SAS 9.4 (SAS Institute Inc., Cary, NC, USA).

## Results

Out of a cohort of 474 KTR, we identified 250 patients meeting inclusion criteria that were furthter categorised into ADNR (*n* = 93), AR (*n* = 22) and C (*n* = 135), as summarized in Table 1. The median time for the first clinically indicated graft biopsy was 22 [10–70] and 13 [7–43] days post-KTx for ADNR and AR, respectively. ADNR group included strictly normal histology (*n* = 53), borderline lesions (*n* = 13), acute tubular necrosis (*n* = 15), recurrent primary disease (*n* = 8), and calcineurin inhibitor (CNI) toxicity (*n* = 4). Therefore, we divided the ADNR group in 3 subcategories: strictly normal histology (*n* = 53), borderline (*n* = 13) and heterogeneous (*n* = 27). AR were cell- (*n* = 14) or antibody-mediated (*n* = 8). Eleven ADNR patients developed AR within the 24-month follow-up period, in a median time of 250 days [93–545] after ADNR diagnosis. Among these 11 cases, two showed borderline lesions at the first clinically indicated biopsy. Only one of ADNR biopsies was exclusively indicated for proteinuria > 1 g/g creatininuria. The mean age (years) at KTx was 50.2 ± 14.2 in ADNR, 47.8 ± 17.8 in AR and 53.6 ± 12.4 in C. The female/male ratio reached 39.8, 45.5 and 34.1% in ADNR, AR and C, respectively. There was no significant difference in the rate of delayed graft function (DGF) among groups. The total number of HLA mismatches was significantly higher in the AR group than in ADNR and C (Table 1).

**Table 1:** Characteristics of the cohort

		C *n* = 135	ADNR *n* = 93	AR *n* = 22	*p*
Recipients	Age (years)	53.6 ± 12.4	50.2 ± 14.2	47.8 ± 17.8	0.07
	Sex Ratio (%F)	34.1	39.8	45.5	0.48
	BMI (kg/m^2^)	25.1 ± 4.4	25.7 ± 5.2	24.2 ± 5.6	0.35
	Dialysis Vintage (days)	581 [260–777]	706 [150–1085]	739 [455–1107]	0.26
	Primary renal disease (%)				0.02
	Diabetic nephropathy	12.6	7.5	13.6	
	Hypertension	4.4	3.2	4.6	
	Glomerulonephritis/vasculitis	26.7	21.5	36.4	
	Cystic/hereditary/congenital	28.2	18.3	22.7	
	Interstitial nephritis/pyelonephritis	3.7	17.2	0	
	Other/unknown etiology	24.4	32.3	22.7	
	PRA Max (0–5 /6–84/85–100%)	80/18.5/1.5	75.3/22.6/2.1	63.6/22.7/13.6	0.13
	PRA > 20% (%)	11.9	10.8	31.8	0.05
Donors	LD (%)	6.7	7.5	22.7	0.09
	DCD (%)	25.4	33.7	29.4	0.42
	Age (years)	43.01 ± 14.5	46.8 ± 12	47.1 ± 12.9	0.08
	Sex Ratio (%F)	45.2	51.6	50	0.62
	BMI (kg/m^2^)	24.4 ± 4.4	25.5 ± 5.2	25.4 ± 5.6	0.18
Transplantation	Re-transplant (%)	17.1	20.7	0	0.05
	CIT (min)	699 ± 314	752 ± 307	582 ± 367	0.06
	HLA MM in toto A + B + DR (n)	2.7 ± 1.1	2.8 ± 1.1	3.5 ± 0.9***	0.02
	Pre-emptive (%)	8.9	18.3	13.6	0.12
	DGF (%)	14.1	26.9	22.7	0.05
Biopsy	Time (days)	95 [91–101]	22 [10–70]***	13 [7–43]***	< 0.01
	Serum Creatinine Levels (mg/dL)	1.3 [1.1–1.53]	2.21 [1.65–3.66]***	2.36 [1.72–3.14]***	< 0.0001
	Proteinuria (mg/g creatininuria)	116 [81–174]	437 [168–992]***	458 [285–1029]***	< 0.001
	CNI (%)	99.3	100	100	0.54
	CsA (%)	9	8.6	13.6	0.78
	MMF derivative (%)	97	97	95.5	0.93
	Oral Steroids (%)	99.3	96.8	91	0.09
	Induction therapy (%)				0.05
	Basiliximab	95.6	93.6	90.9	
	ATG	2.2	3.2	0	
	None	1.5	0	9.1	
	Other	0.7	3.2	0	

*C* controls, *ADNR* acute dysfunction with no rejection, *AR* acute rejection, *BMI* body mass index, *PRA* panel reactive antibody, *LD* living donor, *DCD* donor after circulatory death, *CIT* cold ischemic time, *HLA MM* HLA mismatches, *DGF* delayed graft function, *CNI* calcineurin inhibitors, *CsA* cyclosporine A, *MMF* mycophenolate mofetil, *ATG* antithymocyte globulin. Data are expressed as mean ± standard deviation or as the median and interquartile range (25th–75th percentile). Analysis of variance (ANOVA) was used to compare the 3 groups. ***p < 0.01 (χ^2^ test) versus C

We used a *continuous piecewise linear regression* with a fixed *knot* at 12 months to split the follow-up time into two periods: 0–12 months and 12–24 months. The baseline eGFR value is the average value at 12 months for the intercept of the second part of the regression line. MDRD value at 12 months were 43.5 ± 15.4 mL/min/1.73m^2^ in ADNR, 46.5 ± 15.2 mL/min/1.73m^2^ in AR, and 57.6 ± 14.9 mL/min/1.73m^2^ in C. eGFR at 12 months is significantly different in C versus ADNR (*p* < 0.0001) and AR (*p* < 0.0065), while ADNR and AR are not different from each other (ANOVA, followed by Tukey’s post hoc test). If we analyzed separately the different ADNR subcategories, in comparison with AR and C: MDRD values at 12 months were 38.9 ± 15.5 mL/min/1.73m^2^ in borderline, 47.7 ± 15.5 mL/min/1.73m^2^ in heterogeneous, 42.7 ± 15.2 mL/min/1.73m^2^ in strictly normal histology. eGFR at 12 months is significantly different (*p* < 0.05) in C versus AR, strictly normal and borderline groups, but not versus the heterogeneous group (ANOVA, followed by Tukey’s post hoc test).

The proportion of KTR presenting with a ≥ 30% reduction of eGFR from 12 to 24 months post-KTx reached 16.3% in C versus 29.9% in ADNR (*p* = 0.02) and 15% in AR (not significant). In the ADNR subgroups, this proportion reached 30.8% in borderline group, 34.6% in heterogeneous group, and 27.1% in strictly normal group. There is no statistically significant difference between these subgroups.

**Fig. 1:**
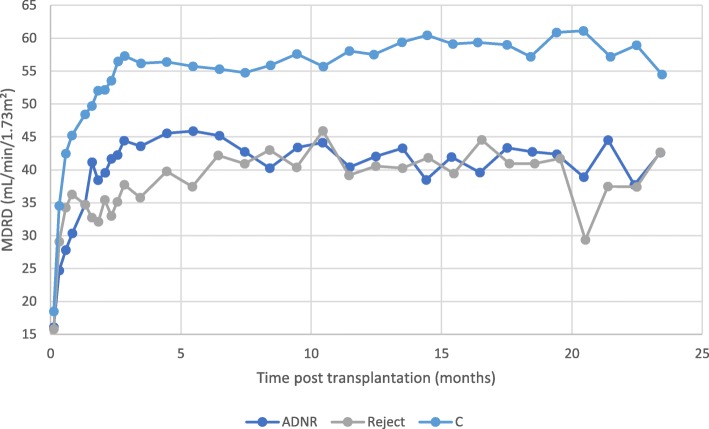
Evolution of MDRD-estimated glomerular filtration rate (eGFR). There are 250 kidney transplant recipients categorized into 3 groups on the basis of histological results of renal graft biopsy: ADNR (acute dysfunction with no rejection, *n* = 93), AR (acute rejection, *n* = 22), C (controls, *n* = 135). The means of MDRD eGFR were exported and plotted against time following kidney transplantation. Data are averaged over 1 week during the first 3 months, then over 1 month until month 24

**Fig. 2:**
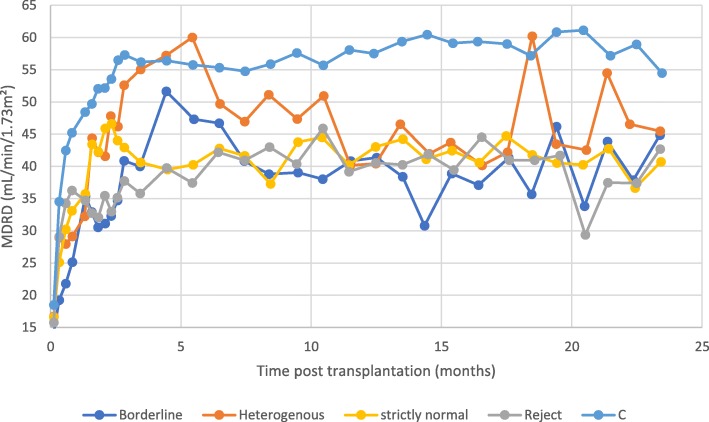
Evolution of MDRD-estimated glomerular filtration rate (eGFR). The 250 kidney transplant recipients categorized into 5 groups on the basis of histological results of renal graft biopsy: borderline (*n* = 13), heterogeneous (*n* = 27), strictly normal (*n* = 53), AR (acute rejection, *n* = 22), C (controls, *n* = 135). The means of MDRD eGFR were exported and plotted against time following kidney transplantation. Data are averaged over 1 week during the first 3 months, then over 1 month until month 24

The evolution of eGFR versus time for the different subgroups (ADNR, AR and C) is presented in Fig. 1, where we calculated weekly averaged eGFR-values for the first 3 months and monthly averaged eGFR-values thereafter. The evolution of time-interval averages of eGFR-values is plotted for the ADNR-subgroups, together with the AR and C subgroup, using weekly averages for the first 3 months and monthly averages thereafter (Fig. 2).

## Discussion

Our analysis shows that ADNR is associated with a significantly lower eGFR at 12 months and greater proportion of patients with a ≥ 30% reduction of eGFR from 12 to 24 months post-KTx in comparison to controls. Kurian S.M. and colleagues originally described the concept of ADNR in the clinical setting of AKI in KTR, thereby distinguishing AR from ADNR by using a three-way instead of the two-way classification algorithm (in which KTR with biopsy-proven AR are classically compared to KTR with stable kidney function) [10]. Doing so, the authors reported on a 38% prevalence of ADNR among their 102-patient cohort. Similarly, Rabant M. et al. [8] categorized their cohort of 281 KTR upon the histological findings of clinically indicated biopsies. These for-cause biopsies were clinically indicated for AKI, proteinuria, identification of de novo DSA, or positive BK virus viremia. Interestingly, the ADNR group involved 203 cases (72.2%) and included acute tubular necrosis/minimal lesions, isolated interstitial fibrosis/tubular atrophy, borderline lesions or a primary diagnosis of recurrent disease. Another work was recently published by Bloom et al. [17] reported a prevalence of 74% of ADNR among 204 clinically indicated biopsies. Similarly to our present data, all of these reports emphasized that ADNR is very frequent in KTR presenting with AKI. Still, the prognosis of ADNR has not been studied thus far. Our observations support that the outcomes of KTR with ADNR appear as poor as AR within the first 2 years *post* KTx, which should prompt specifically dedicated investigations.

The pathophysiology of ADNR entity remains unclear, and most probably multifactorial. In our cohort, kidney graft biopsies were indicated at physicians’ discretion after clinically excluding common causes for AKI in KTR, like dehydration, CNI toxicity or BK virus nephropathy. Note that we found 53 cases with strictly normal histology (57%). From a histological point of view, ADNR is highly heterogeneous. Borderline lesions belong to ADNR group in our series, as well as in previous reports [8, 10]. Using microarray profiling, de Freitas D.G. et al. compared the molecular changes in kidney graft biopsies with (T-cell-mediated) AR, borderline or non-rejection [18]. Most borderline cases showed a molecular phenotype similar to non-rejection, which prompted the authors to substantially question this ambiguous “borderline” category in Banff classification [18]. In a pilot study, our team characterized the putative role of F^18^ fluorodeoxyglucose positron emission tomography coupled with computed tomography (^18^F-FDG PET/CT) in the diagnostic management of KTR with AKI and suspected AR [5]. The mean standard uptake value (SUV) of renal grafts with borderline histology was not statistically different from SUV of control kidneys. Here, only 11 ADNR patients (among whom 2 showed borderline lesions) eventually developed AR, with a median time of 250 days between ADNR and AR biopsies. The low incidence, as well as the long delay between the events, suggests that ADNR and AR are independent nosological entities.

Although the histological and molecular phenotyping of ADNR lesions undoubtedly distinguish it from AR, our present follow-up study demonstrates that KTR with ADNR have poor outcomes within the first 2 post-KTx years. We did not observe significant differences among ADNR subgroups. One may argue that AKI per se is an independent risk factor for chronic kidney disease (CKD), end-stage renal disease (ESRD) and death [19]. Originally considered as fully reversible, AKI is actually associated with permanent kidney damages [19]. Consequently, the diagnostic and therapeutic management of KTR with AKI and suspected AR needs to be significantly improved. AR is one of the main causes of acute kidney graft injury and can be efficiently treated by immunosuppressive drugs. An early detection of AR is therefore essential. It currently depends on the follow-up of serum levels of creatinine, which unfortunately remains an insensitive measure of renal injury. Diagnostic gold-standard ultimately relies on graft biopsy and Banff interpretation [11–13]. Because of cost, risks and sampling errors of biopsies [20, 21], non-invasive approaches are currently under development, like the molecular signature of CD3ε, IP-10 and 18S RNA levels, the urinary biomarkers CXCL9/CXCL10, or imaging techniques [2, 5–10]. These may help avoid unnecessary kidney graft biopsy in patients ultimately presenting with ADNR. Still, in collaboration with non-transplant nephrologists, the transplant community needs to develop innovative therapeutic approaches to prevent the progression from AKI to CKD and ESRD.

Limitations of our present study include its retrospective monocentric design based on a limited number of patients, especially in the AR group (although the relative incidence of AR in our cohort is similar to previous larger series). The use of a surrogate outcome, i.e. ≥30% decline in eGFR within a pragmatic 12-month timeframe, has been previously strongly associated with long-term hard outcomes, including graft failure and death [14]. eGFR decline was shown to be superior to other classical endpoints, like AR occurrence or SCr doubling. Note that, in our present study, MDRD equation was used to estimate GFR, in agreement with previous reports suggesting that MDRD may perform better than CKD-EPI in KTR [22].

## Conclusions

ADNR occurs frequently and early post-KTx, and is associated with poor outcomes in KTR. Prospective research needs to focus on the mechanisms of ADNR in order to improve the diagnostic and therapeutic management of KTR presenting with AKI.

## Data Availability

The datasets used and/or analysed during the current study are available from the corresponding author on reasonable request.

## Abbreviations

ADNR: Acute kidney dysfunction with no rejection
AKI: Acute kidney injury
ANOVA: ANalysis Of VAriance
AR: Acute rejection
CKD: Chronic kidney disease
CKD-EPI: Chronic kidney disease – epidemiology collaboration
CNI: CalciNeurin Inhibitor
DGF: Delayed graft function
DNA: DeoxyriboNucleic Acid
DSA: Donor specific antibody
eGFR: estimated Glomerular Filtration Rate
ESRD: End-Stage Renal Disease
F^18^-FDG PET/CT: F^18^-FluoroDeoxyGlucose Positron Emission Tomography coupled with Computed Tomography
HLA: Human leucocyte antigen
KTR: Kidney transplant recipients
KTx: Kidney Transplantation
MDRD: Modification of Diet in Renal Disease
SCr: Serum Creatinine
SUV: Standard uptake value

## References

[1] Tonelli M, Wiebe N, Knoll G, Bello A, Browne S, Jadhav D (2011). Systematic review: kidney transplantation compared with dialysis in clinically relevant outcomes. Am J Transplant.

[2] Suthanthiran M, Schwartz JE, Ding R, Abecassis M, Dadhania D, Samstein B (2013). Urinary-cell MRNA profile and acute cellular rejection in kidney allografts. N Engl J Med.

[3] Meier-Kriesche HU, Schold JD, Srinivas TR, Kaplan B (2004). Lack of improvement in renal allograft survival despite a marked decrease in acute rejection rates over the Most recent era. Am J Transplant.

[4] Special Issue (2009). KDIGO clinical practice guideline for the Care of Kidney Transplant Recipients. Am J Transplant.

[5] Lovinfosse P, Weekers L, Bonvoisin C, Bovy C, Grosch S, Krzesinski J-M (2016). Fluorodeoxyglucose F 18 positron emission tomography coupled with computed tomography in suspected acute renal allograft rejection. Am J Transplant.

[6] Hanssen O, Erpicum P, Lovinfosse P, Meunier P, Weekers L, Tshibanda L (2017). Non-invasive approaches in the diagnosis of acute rejection in kidney transplant recipients. Part I. in vivo imaging methods. Clin Kidney J.

[7] Erpicum P, Hanssen O, Weekers L, Lovinfosse P, Meunier P, Tshibanda L (2017). Non-invasive approaches in the diagnosis of acute rejection in kidney transplant recipients, part II: omics analyses of urine and blood samples. Clin Kidney J.

[8] Rabant M, Amrouche L, Lebreton X, Aulagnon F, Benon A, Sauvaget V (2015). Urinary C-X-C motif chemokine 10 independently improves the noninvasive diagnosis of antibody-mediated kidney allograft rejection. J Am Soc Nephrol.

[9] Rabant M, Amrouche L, Morin L, Bonifay R, Lebreton X, Aouni L (2016). Early low urinary CXCL9 and CXCL10 might predict immunological quiescence in clinically and histologically stable kidney recipients. Am J Transplant.

[10] Kurian SM, Williams AN, Gelbart T, Campbell D, Mondala TS, Head SR (2014). Molecular classifiers for acute kidney transplant rejection in peripheral blood by whole genome gene expression profiling. Am J Transplant.

[11] Williams WW, Taheri D, Tolkoff-Rubin N, Colvin RB (2012). Clinical role of the renal transplant biopsy. Nat Rev Nephrol Nature Publishing Group.

[12] Racusen LC, Solez K, Colvin RB, Bonsib SM, Castro MC, Cavallo T (1999). The Banff 97 working classification of renal allograft pathology. Kidney Int.

[13] Haas M, Sis B, Racusen LC, Solez K, Glotz D, Colvin RB (2014). Banff 2013 meeting report: inclusion of C4d-negative antibody-mediated rejection and antibody-associated arterial lesions. Am J Transplant.

[14] Clayton PA, Lim WH, Wong G, Chadban SJ (2016). Relationship between eGFR decline and hard outcomes after kidney transplants. J Am Soc Nephrol.

[15] Levey AS, Coresh J, Greene T, Marsh J, Stevens LA, Kusek JW (2007). Expressing the modification of diet in renal disease study equation for estimating glomerular filtration rate with standardized serum creatinine values. Clin Chem.

[16] Weekers L, Vanderweckene P, Pottel H, Castanares-Zapatero D, Bonvoisin C, Hamoir E (2017). The closure of arteriovenous fistula in kidney transplant recipients is associated with an acceleration of kidney function decline. Nephrol Dial Transplant.

[17] Bloom RD, Bromberg JS, Poggio ED, Bunnapradist S, Langone AJ, Sood P (2017). Cell-free DNA and active rejection in kidney allografts. J Am Soc Nephrol.

[18] De Freitas DG, Sellarés J, Mengel M, Chang J, Hidalgo LG, Famulski KS (2012). The nature of biopsies with “borderline rejection” and prospects for eliminating this category. Am J Transplant.

[19] Coca SG, Singanamala S, Parikh CR (2012). Chronic kidney disease after acute kidney injury: a systematic review and meta-analysis. Kidney Int.

[20] Furness PN, Taub N (2001). International variation in the interpretation of renal transplant biopsies: report of the CERTPAP project. Kidney Int Elsevier Masson SAS.

[21] Antonieta Azancot M, Moreso F, Salcedo M, Cantarell C, Perello M, Torres IB (2014). The reproducibility and predictive value on outcome of renal biopsies from expanded criteria donors. Kidney Int.

[22] Masson I, Flamant M, Maillard N, Rule AD, Vrtovsnik F, M-NN P (2013). MDRD versus CKD-EPI equation to estimate glomerular filtration rate in kidney transplant recipients. Transplantation..

